# A Monitoring System for Carbon Dioxide in Honeybee Hives: An Indicator of Colony Health

**DOI:** 10.3390/s23073588

**Published:** 2023-03-29

**Authors:** Martin Bencsik, Adam McVeigh, Costas Tsakonas, Tarun Kumar, Luke Chamberlain, Michael I. Newton

**Affiliations:** School of Science and Technology, Nottingham Trent University, Clifton Lane, Nottingham NG11 8NS, UK

**Keywords:** honeybee, carbon dioxide, CO_2_, humidity, non-dispersive infra-red, NDIR, SCD41

## Abstract

Non-dispersive infra-red (NDIR) detectors have become the dominant method for measuring atmospheric CO_2_, which is thought to be an important gas for honeybee colony health. In this work we describe a microcontroller-based system used to collect data from Senserion SCD41 NDIR sensors placed in the crown boards and queen excluders of honeybee colonies. The same sensors also provide relative humidity and temperature data. Several months of data have been recorded from four different hives. The mass change measurements, from hive scales, when foragers leave the hive were compared with the data from the gas sensors. Our data suggest that it is possible to estimate the colony size from the change in measured CO_2_, however no such link with the humidity is observed. Data are presented showing the CO_2_ decreasing over many weeks as a colony dies.

## 1. Introduction

Water vapor is water in its gaseous state and is called humidity when considered in the air. More often reported as relative humidity, this is the ratio of the partial pressure of water vapor to the equilibrium vapor pressure of water at a given temperature. Temperature and humidity are two of the most important factors affecting the health and survival of honeybee colonies. Humidity is known to play a vital role in the development of brood. Investigations into the effect of humidity were initially made possible by controlled laboratory incubators with more recent data being collected from hives in the field with smaller embedded sensors [1,2,3,4,5,6,7,8,9,10]. Carbon dioxide has long been known as a narcotic for the honeybee and is used to immobilize them during scientific manipulation or transfer. Higher levels of exposure cause permanent damage or result in death. Higher levels in the hive are also believed to initiate fanning behavior. Recent advances in reducing the size of nondispersive infrared measuring devices have resulted in the start of in-hive CO_2_ measurements [11,12,13,14,15,16,17]. Whilst oxygen is clearly important to honeybees, hypoxia is usually tracked indirectly by the measurement of carbon dioxide. Pollutant gases in hives have been reported [18,19,20] but are thought to have less significance to colony health than humidity and carbon dioxide.

The measurement of humidity is readily available in low-cost small capacitive sensors that provide a relevant level of accuracy both in analogue and digital formats. Digital devices usually include temperature measurement in the same package and reduce measurement errors by undertaking the analogue to digital conversion on the sensor chip rather than introducing possible noise in the measurements. Easily deployable breakout boards start for as little as EUR 8 with the raw chips costing even less. Many manufacturers produce these with the inter-integrated circuit (i2c) interfaces [21], each with different addresses allowing some spatial variation over a hive to be monitored with relatively simple and inexpensive hardware.

Beyond humidity, metal oxide semiconductor (MOx) gas sensors provide a range of devices that fall within the size requirements for installation within honeybee hives and in a suitable price range. The problem with these sensors is that most are sensitive to multiple gases, so it is almost impossible to determine which gas is being sensed at any given time. The calibration of a new sensor will also change significantly during the first hours of operation, and it can take several hours to reach a stable value in stable environmental conditions. Care must be taken to read the datasheets as the advertising may suggest specificity whereas they merely have somewhat higher sensitivity to one target gas. The basic arrangement comprises a heater circuit and a measurement circuit where the change of conductivity in the MOx layer is measured. A further consideration is the required power supply voltage with most modern microcontrollers operating from 3.3 V, some of the older sensors use a 5 V supply adding additional barriers to implementation and higher power consumption as a result.

One family of these MOx sensors respond to give an estimate for CO_2_, typically derived from hydrogen measurement and termed eCO_2_ or CO_2_eq along with a value that represents the total volatile organic compounds (TVOC). These together can produce a measure of air quality that is mainly aimed at indoor environments. Different manufacturers produce such sensors with the most popular being the SGP30 [22] (Sensirion AG, Stäfa, Switzerland), the iAQ-CORE [23] (AMS AG, Unterpremstaetten, Austria), and the CCS811 [24] (Sciosense B.V., Eindhoven, The Netherlands). The BME680 [25] (Bosch Sensortec GmbH) is similar but produces a single air quality index from the gas sensor and also returns humidity, pressure, and temperature. All of these use the i2c interface and suitable driver routines are available for microcontrollers such as the Arduino or Python drivers for the Raspberry Pi. They can be purchased ready assembled onto breakout printed circuit boards; however, it is worth going for one of the well-known brands such as Adafruit [26] and Sparkfun [27] who provide well tested driver routine for their boards to make programming relatively simple for around EUR 25 per sensor board. Typical power consumption for these is between 20 and 80 mW, making them suitable for leisure battery powered field deployment. Comparisons of these have been published by Yurko 2019 [28], Lasomsri 2018 [29], and Arroyo 2020 [30]. Whilst straightforward to code, particularly with the branded expansion boards, these articles highlight the requirement for calibration prior to deployment.

For calibrated CO_2_ measurement the nondispersive infrared (NDIR) sensor is the closest sensor to meeting the requirements for hive installation. The basic principle is that an infrared (IR) source, closely matched to the absorption frequency of CO_2_, shines down a sample tube containing air. The nondispersive terminology comes from there being no grating or prism to select the frequency of light, rather, at the end is a filter to remove other frequencies followed by an IR detector. The difference between the amount of light radiated by the IR source and amount of IR detected is directly proportional to the number of CO_2_ molecules in the air sample in the tube. Unlike MOx sensors, this is specific to CO_2_ and can be calibrated to directly measure the concentration of CO_2_ in ppm. However, commercial devices tend to be larger than the MOx sensors, typically 35 mm × 25 mm × 7 mm, meaning that they will need to be mounted either in a hive wall, or replace part of the comb, but are similar in size to a typical queen cage. The cost is also greater than MOx, typically EUR 70. Suitable models include the Sensirion (AG, Stäfa, Switzerland) SCD30 [31], which also includes temperature and humidity, the Teledyne T6713 [32] (Amphenol Thermometrics, Inc., St. Marys, PA, USA), and the Figaro CDM7162 [33] (Figaro Engineering Inc., Osaka, Japan). A lower cost alternative is the MH-Z19B [34] (Zhengzhou Winsen Electronics Technology Co., Ltd., Zhengzhou, China), but this does not include an i2c and has slightly lower range than the others. These devices are also not instant ‘plug and play’ for calibrated measurements as, on first use, several days are often required as burn in and are recommended to need some 30 min to give accurate readings. However, once running, these devices do give actual CO_2_ gas concentration in parts per million within their specified error margin, typically 30 ppm +3%. A recent variation [35] uses the photoacoustic NDIR sensing principle to reduce the physical size, this is the Sensirion SCD41 (Sensirion AG, Stäfa, Switzerland).

Over the years, numerous studies have shown the importance of different gases to the health of honeybee colonies, however, much of the data have used expensive laboratory-based analysis or controlled environments. The lack of availability of low cost, small, and highly specific gas sensors continue to be a limitation in this area of study. For now, the reasonably accurate measurement of humidity along with NDIR measure of CO_2_ are the most that can reliably be achieved on a budget for continuous in-hive monitoring. In this article, we report the use of the Sensirion SCD41 for application in honeybee hives as these provide a CO_2_ measurement range of up to 40,000 ppm, with a specified accuracy of ±40 ppm +5% of reading up to 5000 ppm, in addition to providing humidity and temperature data.

## 2. Materials and Methods

**Figure 1:**
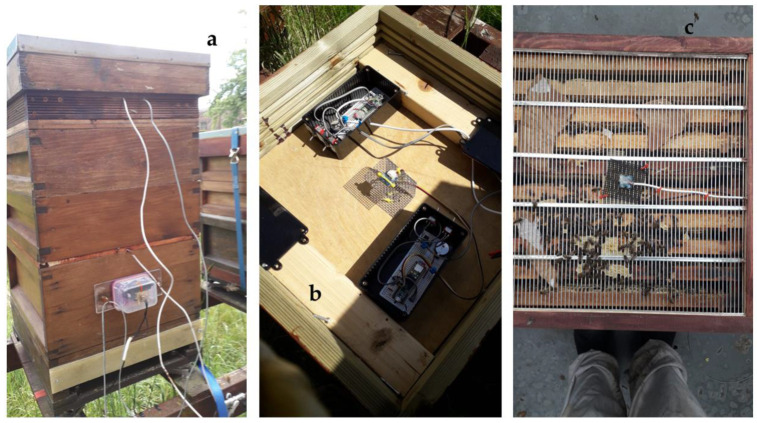
(**a**) Hive showing brood box with queen excluder, two ‘supers’, and the modified crown board. (**b**) Modified crown board with two sets of measurement electronics and one SCD41 sensor mounted over a hole in the center. (**c**) Queen excluder with SCD41 sensor and protective mesh cage.

The honeybee colonies with gas sensor systems were sited at both the Nottingham Trent University Clifton campus in the UK and the nearby B-GOOD apiary at Holme Pierrepont Hall. All data presented here are from Holme Pierrepont hives 5, 6, 7, and 8 (referred to below as HPP5, HPP6, HPP7, and HPP8). The basic structure of a honeybee hive consists of the brood box, in our case the British National hive brood box, in which the queen lays eggs and the young are reared. A queen excluder is placed on top on the brood box to prevent the queen from moving to the upper ‘super’ boxes above the brood box but allowing workers to pass through. The supers are predominantly where the honey is produced and stored. Additional supers can be added through the season as the supers become full or full frames of honey replaced with empty frames. On top of the supers is a crown board and, in this work, we produced a modified crown board that could hold the measurement electronics and the uppermost gas sensor in a weatherproof environment. Figure 1a shows one of the hives with a brood box and two supers below the modified crown board. Figure 1b shows the inside of the modified crown board with the two sets of measurement electronics and a gas sensor mounted over a mesh covered hole in the center. A second gas sensor was mounted on the queen excluder, and this is shown in Figure 1c with the protective mesh that somewhat protects it from propolis and wax coating by the bees. The hives were also mounted on a BEEP hive scale [36] that provided the mass of the hive every 15 min; although the BEEP system measures other parameters, only the hive mass data were used in this work.

**Figure 2:**
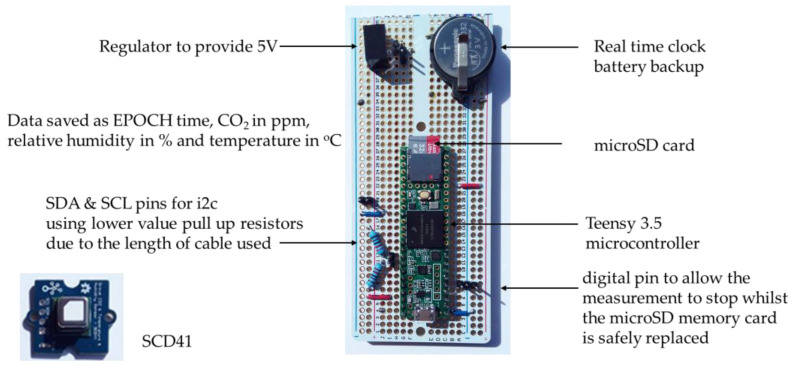
Data logging electronics based on Teensy 3.5 microcontroller. The SCD41 NDIR sensor is shown bottom left mounted on the Grove breakout board.

The Teensy 3.5 microcontroller (PJRC, Sherwood, OR, USA) was chosen as the data logging system as it provided a real-time clock, with a battery backup, and microSD card reader on board (Figure 2). The gas sensors were connected to the Teensy via the i2c bus; a switch on one digital pin allowed the measurement to stop whilst the microSD memory card was safely replaced. As cable lengths to the sensors were at the upper end of that permitted in i2c, the pull up resistor values were dropped from 4.7 to 2.2 kΩ. Data consisting of EPOCH time (in seconds), CO_2_ (in ppm), temperature (in °C), and relative humidity (as a percentage) were saved with around three measurements per minute to approximately one file per day on the microSD card. The EPOCH (or Unix timestamp) is the number of seconds that have elapsed since 1 January 1970, and using this format allows data from different systems to be directly compared with each other. Reliability has proved excellent as all data presented in this article are as measured with dropped measurements showing as zeros in the data. With only a handful of data points dropped a month, this represents less than one in 25,000 measurements. The SCD41 gas sensors were purchased as Grove breakout boards from Seeed Technology (Shenzhen, China) [37] as these provided a more robust mechanism for soldering the wire connections compared to just the SCD41 element. The socket was removed from the rear of the Grove circuit board to save space as the same connections were also provided through hole solder terminals.

## 3. Results

**Figure 3:**
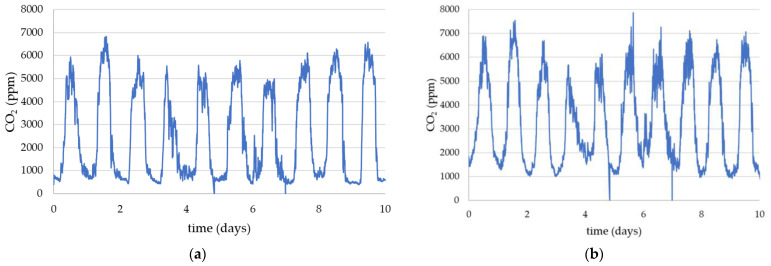
CO_2_ data from HPP8 measured at a sensor in the crown board (**a**) and the queen excluder (**b**) covering the same measurement times.

Figure 3 shows the CO_2_ measured in the HPP8 colony from 8 July 2022 to 18 July 2022 with (a) the crown board sensor data and (b) the queen excluder sensor data. Taking day 6 data as an example for the crown board (14 July 2022), the CO_2_ starts to fall rapidly from 5000 ppm at 6 am and reaches the minimum at 3.25 pm of less than 1000 ppm. This is followed by variations and a slow rise until the CO_2_ starts to rise rapidly again at 10 pm to 4500 ppm with variations of around 500 ppm until the next day at 7 am. Figure 3 data, collected in July, represent some of the longest hours of daylight and hence the longest foraging time; this can be seen to reduce the CO_2_ data as daylight hours reduce going into autumn.

**Figure 4:**
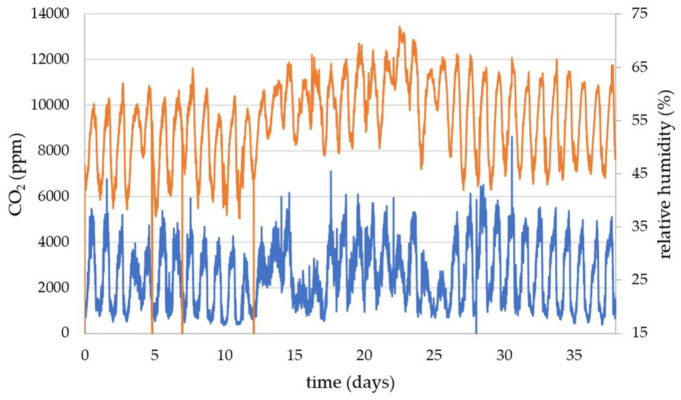
HPP6 queen excluder sensor comparing the CO_2_ (blue data using left hand side axis) with the relative humidity (orange data using right hand axis).

Figure 4 shows the CO_2_ and relative humidity from the HPP6 queen excluder sensor covering the period 8 July 2022 to the 15 August 2022. The daily variation can be seen in both CO_2_ and relative humidity and, in this particular data set, the daytime peak in the relative humidity tend to correspond to the peak in the CO_2_. However, this is not the same for all data and during some periods the daily changes in relative humidity were not seen to be directly aligned with the CO_2_ variation. Relative humidity is strongly dependent on the ambient weather conditions, which can make the changes in relative humidity due to the honeybee colony a small perturbation on the overall relative humidity value. A detailed study of the relative humidity is beyond the scope of this current article.

## 4. Discussion

The most obvious feature from Figure 3 is that the rise and fall in CO_2_ data occur at the same times during the day in both the crown board and the queen excluder. However, the CO_2_ falls during the daytime to almost ambient air values (450 to 500 ppm) in the crown board but is around twice this value in the queen excluder. This is not unexpected as the queen excluder sensor is mounted close to the bulk of the colony. The change between night and day is consistent with previous recent reported values [10,15,16,17] and these changes in CO_2_ are similar, if not in absolute values, in both sensor locations.

**Figure 5:**
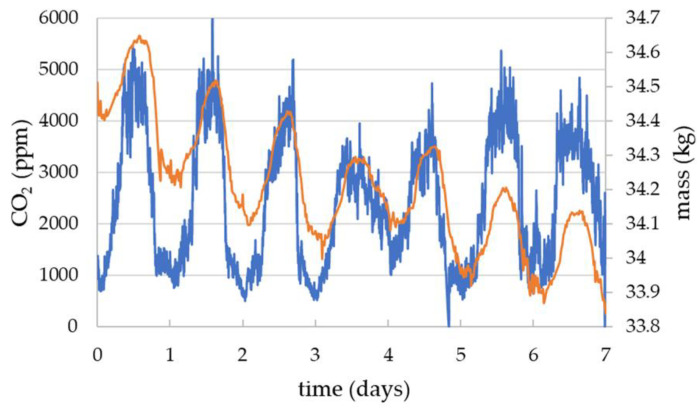
HPP6 queen excluder sensor CO_2_ (blue data using left hand axis) compared with the hive mass (orange data using right hand axis) from the BEEP scale. The daytime exit and return of the foragers can clearly be seen in both the mass and the CO_2_ data.

**Figure 6:**
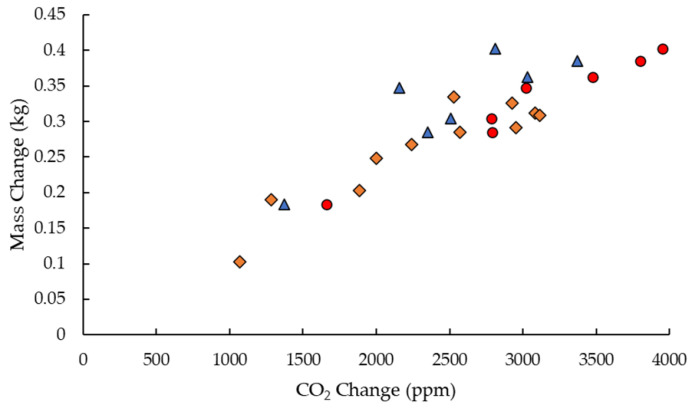
Using data similar to that shown in Figure 5, daytime change in mass due to foragers leaving the hive is plotted against the change in CO_2_ at the same time. Data for three hives sensors are shown: HPP8 crown board (diamonds), HPP6 queen excluder (circles), and HPP6 crown board (triangles).

The data in Figure 5 are representative of the correlation between the changes in mass and CO_2_ through the day. During periods of nectar flow there is a significant back slope imposed on the daily increase and decrease in the mass data and these data did not allow accurate measurements of the daily changes specifically due to the foragers. However, Figure 6 shows data for periods similar to that shown in Figure 5, where mass changes are predominantly due to exit and return of foragers, and has allowed the daily change in mass to be plotted against the daily change in CO_2_ for three different sensors.

**Figure 7:**
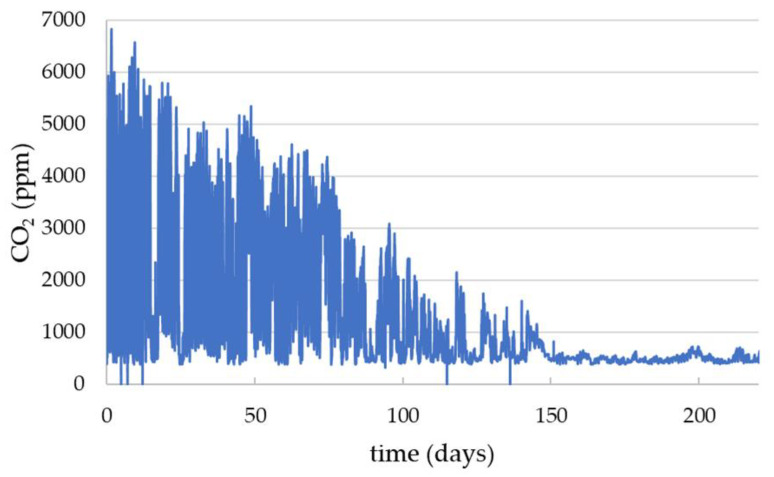
HPP8 crown board CO_2_ data from 8 July 2022 to 14 February 2023. Inspections found that this colony was healthy at the start of the period and was observed as failed at around 150 days.

From the gradient of Figure 6, we can determine that the CO_2_ change is (8701 ± 928) ppm per kg of bees. A simple calculation using the average weight of a worker honeybee being 120 mg [38,39] gives (1.04 ± 0.10) ppm/bee. This cannot be directly ascribed to the physiological activity as the physical presence of the forager bees will also change the air flow in the hive. However, this suggests that it may provide an empirical way to estimate the colony size from CO_2_ changes when the forages leave the hive and without the requirements for a hive scale. This is clearly demonstrated in the tracking of the decline of colony HPP8 shown by the CO_2_ data in Figure 7. This covers the period 8 July 2022, when the colony was healthy, through to the 14 February 2023. The CO_2_ follows a ‘healthy’ trend up until 29 July after which there is a steady decline in the daily CO_2_ variation and colony death by 150 days after which the CO_2_ levels are consistent with ambient air.

## 5. Conclusions

The current generation of NDIR carbon dioxide sensors have been demonstrated to be well suited to the task of monitoring in honeybee colonies. We have shown preliminary data that suggests that CO_2_ can give information about the number of foraging bees in the hive and hence the colony strength and has shown how this decreases in a dying colony. The very high temporal resolution that we have used has produced excellent data, but the colony demise could equally well have been determined from measurements as low as four per hour which is typical of commercial hive monitoring scales. With the ubiquitous i2c interface being used by most NDIR sensors in this class, it would be straightforward for manufacturers of hive scales to incorporate such devices into their hardware. One simple technique we have developed is the modified crown board for holding electronics which has proven to be outstanding for keeping the electronics dry and functioning. As the CO_2_ sensor gives good data from this location, it may be something else that hive monitoring equipment manufacturers may wish to consider implementing for themselves.

The next stage of this research will be to look at CO_2_ in hives during the wintertime when the brood boxes may not be opened. As little data are currently available on wintering colonies, CO_2_ may give an important indication of when a colony may require extra feeding to survive. Further studies may also include the comparison of CO_2_ in colonies in the same location but with differing levels of varroa mite infestation to see if CO_2_ offers any indication of colony varroa levels.

## Data Availability

The data presented in this study are openly available in FigShare at https://figshare.com/articles/dataset/Gas_sensor_data_from_summer_2022/22133651 or doi:10.6084/m9.figshare.22133651 (accessed on 25 March 2023).
